# Issues and prospects of image‐guided thermal ablation in the treatment of primary and metastatic lung tumors

**DOI:** 10.1111/1759-7714.14742

**Published:** 2022-12-08

**Authors:** Meixiang Wang, Zhigang Wei, Xin Ye

**Affiliations:** ^1^ Department of Oncology The First Affiliated Hospital of Shandong First Medical University & Shandong Provincial Qianfoshan Hospital, Shandong Key Laboratory of Rheumatic Disease and Translational Medicine, Shandong Lung Cancer Institute Jinan Shandong Province China; ^2^ Cheeloo College of Medicine Shandong University Jinan Shandong Province China

**Keywords:** early‐stage, image‐guided thermal ablation, issues and prospects, non‐small cell lung cancer, pulmonary metastases

## Abstract

The precise local minimally invasive or noninvasive treatment represents the important orientation for advancing the treatment of pulmonary malignant tumors. New local treatment methods have emerged as solutions to the shortcomings of minimally invasive or local treatment methods. Image‐guided thermal ablation (IGTA) comes with the characteristics such as more accurate localization, less trauma, more definite efficacy, higher safety, stronger repeatability, fewer complications, and lower cost in treating lung tumors. This paper investigates the existing problems of IGTA in the treatment of lung tumors and puts forward the orientation of studies.

## INTRODUCTION

With the development of modern imaging and the prevalence of health screening, the early detection rates of primary lung cancer and lung metastasis have increased significantly. Early diagnosis and treatment are the keys to influencing the prognosis of patients with primary non‐small cell lung cancer (NSCLC) and lung metastases (especially oligometastases). Therefore, locally precise, minimally invasive, or noninvasive treatment is an important direction in treating lung tumors. The wide application of minimally invasive surgery represented by video‐assisted thoracoscopic surgery (VATS) and noninvasive therapy represented by stereotactic body radiation therapy (SBRT) has improved the therapeutic efficacy of lung tumors; however, both VATS and SBRT have certain limitations.^1^, ^2^, ^3^ Therefore, many novel local treatment approaches have been developed, including image‐guided thermal ablation (IGTA) therapy. IGTA mainly includes radiofrequency ablation (RFA), microwave ablation (MWA), cryoablation, and laser ablation. IGTA, a precise minimally invasive technique, has been applied to treat early‐stage lung cancer and pulmonary metastases. Moreover, the number of patients with lung tumors treated using IGTA each year is increasing rapidly.^4^, ^5^, ^6^, ^7^, ^8^, ^9^, ^10^

## CLINICAL OUTCOMES

### Early‐stage lung cancer

From the current literature review,^11^, ^12^, ^13^, ^14^, ^15^, ^16^ we noted that for patients with early‐stage NSCLC whose tumor diameter was <3 cm and medically inoperable surgical resection, the 1‐, 3‐, and 5‐year overall survival (OS) rates after IGTA reached 89%–95.7%, 68%–72.9%, and 20%–61.7%, respectively, and the perioperative mortality was <1%. Furthermore, for pulmonary oligorecurrence after radical resection of early‐stage NSCLC, MWA has also achieved good clinical efficacy. In a recent multicenter study, the median OS of patients with pulmonary oligorecurrence after radical resection of early‐stage NSCLC who were treated with MWA was 40.6 months.^17^

Chan et al.^5^ reported the meta‐analysis results of IGTA and surgical resection in the treatment of early‐stage lung cancer (tumors diameter of ≤2 cm). The median OS rates of patients undergoing IGTA versus surgical resection were 45.8 versus 49.2 months, respectively; the 1‐, 3‐, and 5‐year survival rates were 92, 62, and 41% versus 95, 76, and 49%, respectively; the 1‐, 3‐, and 5‐year tumor‐specific survival rates were 94%, 72%, and 60% versus 94, 76, and 68%, respectively; there were no significant differences in the three indexes of survival.

Uhlig et al.^7^ analyzed the National Cancer Database of the United States and compared the results of IGTA and SBRT in patients who could not tolerate surgical resection of early‐stage NSCLC. The 1‐, 3‐, and 5‐year survival rates were 85.4, 47.8, and 24.6% versus 86.3, 45.9, and 26.1%, respectively. There was no significant difference. Several recent reports^2^, ^18^, ^19^ support the above view.

### Lung metastases

Pulmonary metastatic disease is experienced commonly in colorectal cancer (CRC), sarcoma, melanoma, head and neck cancers, breast cancer, and tumors of the urinary tract. Numerous other malignancies are also known to spread to the lungs. For appropriately selected patients, the National Comprehensive Cancer Network guidelines suggest the consideration of ablative techniques, provided that all visible disease is eradicated. IGTA offers the benefit of being able to safely treat metastases while preserving normal pulmonary parenchyma and function. IGTA has been shown to be a technically feasible and relatively safe treatment option for patients with new as well as recurrent pulmonary metastases, with a preponderance of the existing data in the area of colorectal metastases.^2^ In a review by Delpla et al. the local control rate was 62%–91%, the 5‐year survival rate was 32%–65%, and the median survival was 35–70 months.^20^ Therefore, Cao et al.^21^ believed that the clinical efficacy of IGTA in treating lung oligometastasis of CRC might be better than that of SBRT.

## LIMITATIONS AND PROSPECTS

### Prospective, multicenter, randomized, and controlled clinical trials

According to the current clinical data on IGTA in the treatment of early NSCLC, most studies are retrospective in nature and the level of evidence‐based medicine is low.^13^ There is still a lack of prospective, multicenter, randomized, and controlled clinical trials on whether IGTA can become one of the primary treatments for patients with early‐stage NSCLC. Among them, prospective, multicenter, randomized, and controlled trials of IGTA, surgical resection, and SBRT are the difficulties and also the focus of future work.

As low‐dose computed tomography (LDCT) screening programs have been widely conducted in recent years, many early‐stage ground‐glass nodule (GGN)‐like lung cancer have been detected. However, GGN‐like lung cancer is characterized by “indolent” development, with distant metastasis in very few patients; the disease has a favorable prognosis and a 100% 5‐year survival rate after VATS. GGN‐like lung cancer is a special subtype of lung cancer as it differs from traditional early‐stage lung cancer. The following problems exist in the premature use of VATS to remove this type of lesion: (1) Premature surgical intervention for pulmonary nodules, particularly precancerous lesions, will lead to early and unnecessary organ damage and lung function loss. Moreover, early surgery cannot significantly improve the OS of patients when compared with those whose interventions are follow‐up and elective surgery. (2) There are no clear selection criteria for surgical intervention of multiple pulmonary nodules and no principles for the follow‐up management of residual nodules. (3) Preoperative diagnosis of pulmonary nodules is made using imaging without pathological evidence. Surgical resection of pulmonary nodules may be unnecessary and causes postprocedural complications when the lesions are found to be benign. (4) As the population ages, many patients with early‐stage lung cancer are diagnosed above the age of 75 years, when surgery is almost impossible. Moreover, there are problems with follow‐up, such as follow‐up intervals and termination. Each re‐examination may trigger anxiety, affect quality of life, and increase the patient's exposure to X‐rays. New approaches to manage lung nodules must be explored to solve the above problems. IGTA also shows advantages in treating GGN‐like lung cancer,^22^, ^23^, ^24^, ^25^ which is one of the main developmental directions of treatment, but prospective, multicenter, randomized, and controlled clinical trials are needed.

### Homogenization

IGTA is an emerging technology, and employees' professional and technical levels in this field are uneven. It is difficult to homogenize IGTA technology owing to the different equipment used in each medical institution. Therefore, it is necessary to establish and improve the standardized training system, professional team, and case registration management system for IGTA. In addition, puncture robots, navigation systems, 3D printing technology, and artificial intelligence planning and validation systems are likely to be helpful in the homogenization of IGTA technology.

### Local response

As a local treatment technique, IGTA has two major problems: “how to improve complete ablation” and “how to reduce postprocedural local progression (or recurrence).” For example,^26^ before 2011, the 3‐year local progression rate for microwave ablation for early lung cancer was 22%–37%; however, after 2011, it was 9%–26%, thus showing a downward trend. Accurate patient selection, especially based on tumor size and location, is important in improving complete ablation and reducing postprocedural progression. The complete ablation was significantly higher and the local progression was significantly lower in patients with a tumor diameter of <2 cm than in those with a diameter of ≥2 cm. It is difficult to completely ablate tumors >3 cm using IGTA. Owing to the relatively few vessels and bronchus in the outer one third of the lung parenchyma, the complete ablation of tumors in the region was significantly higher than that in the middle and inner one third of the lung parenchyma; furthermore, the local progression rate was also significantly lower than that in the middle and inner one third.

The ablative margin is also key to improving complete ablation and reducing postprocedural progression. When the ablative margin beyond the tumor was ≥5 mm (ideally 10 mm), complete ablation was significantly higher than that of a tumor with an ablative margin of <5 mm; local progression was also significantly lower than that of a tumor with an ablative margin of <5 mm.^27^ However, excessive ablation may occur if the ablative margin is emphasized too much. An ablative margin beyond the tumor of >10 mm can aggravate lung parenchymal injury and cause more complications.

### Efficacy evaluation criteria

Efficacy evaluation after IGTA is a clinical problem to be solved urgently. The current Response Evaluation Criteria in Solid Tumors are based on the maximum tumor diameter and are not suitable for efficacy evaluation after IGTA. It is sometimes difficult to ascertain the efficacy of IGTA using the imaging methods.^24^ Therefore, there is a need for long‐term work to formulate widely recognized efficacy evaluation criteria in line with the characteristics of IGTA technology itself. In addition, we should break through the efficacy evaluation mode of an imaging method, and it is necessary to establish a mode of combined efficacy evaluation based on both biomarkers and imaging. Therefore, searching for biomarkers with high sensitivity and specificity is one of the future research directions in efficacy evaluation.

### Combination with other treatments

There are few clinical studies on the combined use of IGTA and other treatment methods (such as radiotherapy, chemotherapy, molecularly targeted drugs, and immunotherapy).

For advanced NSCLC, IGTA combined with chemotherapy provides certain benefits, such as improving the local control rates of tumors and prolonging the survival of patients.^28^, ^29^, ^30^, ^31^ Therefore, this combination may be used as a new approach for treating advanced‐stage NSCLC.

Tyrosine kinase inhibitors (TKIs) are currently one of the main approaches used for treating NSCLC with *EGFR* mutations or ALK‐EML4 fusion mutations. The administration of TKIs in such patients can achieve an objective response rate of approximately 70% and progression‐free survival (PFS) of approximately 10–30 months. However, most patients ultimately develop acquired resistance to TKIs after 1–1.5 years. Therefore, it is important to distinguish among these patterns as different therapeutic strategies may apply. Considering the growth rate of the tumor and the number of growing tumor lesions, progressive disease during TKI treatment can be generally distinguished into three patterns: intracranial disease progression, development of one or few distant metastatic sites while the patient remains asymptomatic, and systematic and/or symptomatic disease progression. The first two patterns fall under the general category of oligoprogressive disease, which refers to the presence of less than five discrete metastatic sites. Local ablation with continued EGFR inhibition has shown efficacy in treating patients with oligoprogressive disease and is associated with long PFS and OS.^32^ Local ablation with continued TKI treatment can be used as a treatment strategy for advanced‐stage NSCLC in which extracentral nervous system oligoprogressive disease has developed during EGFR TKI treatment.^33^

After IGTA, tumor tissues can release several tumor‐related antigens, which help enrich many T cells locally and alter the tumor microenvironment. Therefore, the combination of immunotherapy with IGTA is theoretically feasible. At present, there are also a few reports on IGTA combined with immunotherapy in the treatment of advanced NSCLC,^34^, ^35^, ^36^, ^37^, ^38^, ^39^, ^40^, ^41^ which suggests that the two exhibit a synergistic effect. Therefore, IGTA combined with immunotherapy in the treatment of advanced NSCLC is likely to become a research hotspot in the future.

### Neoadjuvant and adjuvant therapy

In the surgical field, neoadjuvant chemotherapy/immunotherapy/TKI therapy with reduced clinical stage, followed by surgical treatment and sequential adjuvant chemotherapy/immunotherapy/targeted drug therapy, has become a new model of possible cure for cancer. For example, for patients with stage II–IIIA NSCLC with a tumor diameter of ≥5 cm or even ≥7 cm and no lymph node metastasis, the tumor diameter is reduced to ≤3 cm with neoadjuvant chemotherapy/immunotherapy/targeted drug treatment, followed by IGTA treatment and sequential adjuvant chemotherapy/immunotherapy/TKI therapy. This mode of “neoadjuvant therapy + IGTA + adjuvant therapy” is likely to become one of the research directions in the future. In addition, for patients with high‐risk factors, EGFR or ALK‐EML4 fusion mutations, or PD‐L1 expression of >50% who undergo IGTA for early lung cancer, adjuvant chemotherapy/immunotherapy/TKI therapy to reduce recurrence and metastasis is likely to become one of the key research directions in the future.

### Palliative ablation

Palliative ablation is one of the important components in the comprehensive treatment of tumors. The purpose of palliative thermal ablation is to relieve symptoms caused by the tumor, improve the patient's quality of life, and prolong the lifespan as much as possible. It is better to decide the indications for palliative ablation after multidisciplinary team (MDT) discussion. For example, in case of refractory pain caused by tumor invasion into the ribs or vertebral body, ablation can be performed at the local bone invaded by the tumor (or combined with other treatments, such as the use of bone cement) to achieve analgesic effects.^42^, ^43^, ^44^

### Basic research

At present, there is a basic lack of research on IGTA. The distribution and monitoring of complex thermal fields and biothermodynamics need to be studied in the future. Recent studies have found that in addition to necrosis and apoptosis, a new form of cell death, “pyroptosis,” is caused by thermal ablation.^45^, ^46^, ^47^, ^48^, ^49^ The emergence of the pyroptosis concept is likely to clarify the exact mechanism underlying tumor cell death caused by thermal ablation, and studies on the relationship between thermal ablation and pyroptosis are needed. There is a further need to explore the effect of thermal ablation on the immune function, which remains unclear.

### Others

With the emergence of nanomaterials, nanothermal sensitizers and contrast agents can be continuously developed and applied in clinical applications. New guidance methods, such as electromagnetic navigation technology,^50^, ^51^, ^52^, ^53^ are gradually being developed for the thermal ablation treatment of lung cancer. These methods show certain advantages in improving precision ablation, but their popularization remains difficult. Moreover, a lot of work needs to be done to intelligent and upgrade ablation equipment, such as continuous improvement of microwave antennas. Temperature control is also a significant issue in the treatment of MWA. With the application of preoperative three‐dimensional visualization planning system and the temperature surveillance periprocedure, a more precise temperature control may be achieved.

## CONCLUSIONS

In conclusion, as an independent local treatment technology for tumors, IGTA has become the third major local therapeutic method for tumors, following surgery and SBRT. IGTA is likely to be more and more widely used in the comprehensive treatment of lung tumors. However, there are certain limitations associated with the use of IGTA techniques for treating lung tumors. The possibilities of combining IGTA with systemic chemotherapy, molecularly targeted drug therapy, and immunotherapy needs to be investigated deeply in the future.

## AUTHOR CONTRIBUTIONS

Meixiang Wang: Data collection and writing. Zhigang Wei and Xin Ye: Concept, design, writing confirm.

## FUNDING INFORMATION

This study received funding from National Natural Science Foundation of China (81502610 and 82072028) and Natural Science Foundation of Shandong Province, China (ZR201911040313).

## CONFLICT OF INTEREST

The authors declare no competing interests.
